# Detection of Recombinant African Swine Fever Virus Strains of p72 Genotypes I and II in Domestic Pigs, Vietnam, 2023

**DOI:** 10.3201/eid3005.231775

**Published:** 2024-05

**Authors:** Van Phan Le, Van Tam Nguyen, Tran Bac Le, Nguyen Tuan Anh Mai, Viet Dung Nguyen, Thi Tam Than, Thi Ngoc Ha Lai, Ki Hyun Cho, Seong-Keun Hong, Yeon Hee Kim, Tran Anh Dao Bui, Thi Lan Nguyen, Daesub Song, Aruna Ambagala

**Affiliations:** Vietnam National University of Agriculture, Hanoi, Vietnam (V.P. Le, N.T.A. Mai, V.D. Nguyen, T.T. Than, T.N.H. Lai, T.A.D. Bui, T.L. Nguyen);; Institute of Veterinary Science and Technology, Hanoi (V.T. Nguyen);; Chungnam National University, Daejeon, South Korea (T.B. Le);; Animal and Plant Quarantine Agency, Gimcheon, South Korea (K.H. Cho, S.-K. Hong, Y.H. Kim);; Seoul National University, Seoul, South Korea (D. Song);; National Centre for Foreign Animal Disease, Winnipeg, Canada (A. Ambagala).

**Keywords:** African swine fever, African swine fever virus, Vietnam, swine, viruses, pigs

## Abstract

African swine fever virus (ASFV) genotype II is endemic to Vietnam. We detected recombinant ASFV genotypes I and II (rASFV I/II) strains in domestic pigs from 6 northern provinces in Vietnam. The introduction of rASFV I/II strains could complicate ongoing ASFV control measures in the region.

African swine fever is a devastating and lethal swine disease that poses a major threat to wild boars and domestic pigs (*1*). The causative agent, African swine fever virus (ASFV), belongs to the Asfaviridae family and has a double-stranded DNA genome with a length of ≈170–190 kb (*2*). The ASFV genome encodes >150 proteins. ASFV strains have been classified into 24 genotypes on the basis of the C-terminal sequence of the B646L gene, which encodes the p72 protein (*3*). Recently, a new classification based on the complete protein sequence of p72 was proposed that would classify ASFV strains into 6 genotypes (*4*). ASFV of the p72 genotype II first emerged in China in 2018, then spreading to nearby countries in Asia, including Vietnam, Myanmar, South Korea, Indonesia, the Philippines, Cambodia, India, and Bangladesh. In 2021, China reported the detection of low-virulence genotype I ASFV strains (Pig/HeN/ZZ-P1/2021 and Pig/SD/DY-I/2021) with high genetic similarity to the nonhemadsorbing strains NH/P68, isolated in 1968, and OURT88/3, isolated in 1988, both from Portugal (*5*). Several attenuated, low-virulence p72 isolates of genotype II have also been reported from China (*6*). 

In 2023, China reported the emergence of highly virulent recombinant ASFV strains of genotypes I and II (rASFV I/II) from Jiangsu (JS/LG/21) and Henan (HeN/123014/22) Provinces and Inner Mongolia (IM/DQDM/22) that show resistance to immunity induced by HLJ/18–7GD, a 7-gene deleted live-attenuated ASFV genotype II vaccine (*7*). The first outbreak of African swine fever in Vietnam was reported in Hung Yen Province in 2019 (*8*). The responsible virus was later identified as a highly pathogenic p72 genotype II strain that was like the strains circulating in China at that time. Because China announced the discovery of the ASFV p72 genotype I, II, and rASFV I/II strains, we have conducted a rigorous surveillance program focused on the northern provinces of Vietnam to monitor emerging ASFV strains (*9*,*10*).

## The Study

In September and October 2023, we collected a total of 26 whole blood samples from pigs suspected to be infected with ASFV from family farms in 6 different northern provinces of Vietnam (Hai Duong, Bac Giang, Hanoi, Phu Tho, Tuyen Quang, and Thai Nguyen) (Appendix Figure 1). We tested the samples by using an ASFV-specific real-time PCR (Median Diagnostics Inc., https://mediandiagnostics.com), according to the kit instructions. Our results showed that all samples were positive for ASFV; cycle threshold values ranged from 16.58 to 32.13 (data not shown). We then tested the samples by using conventional PCR to amplify the C-terminal region of B646L (p72) and the full-length sequences of the E183L (p54) and EP402R (CD2v) genes, followed by Sanger sequencing (1st BASE, https://base-asia.com) (*11*). We assembled and analyzed the sequences by using Geneious Prime (Geneious, https://www.geneious.com), and we performed the alignment of nucleotide and amino acid sequences by using BioEdit version 7.7.1.0 (https://thalljiscience.github.io/page2.html).

**Figure 1:**
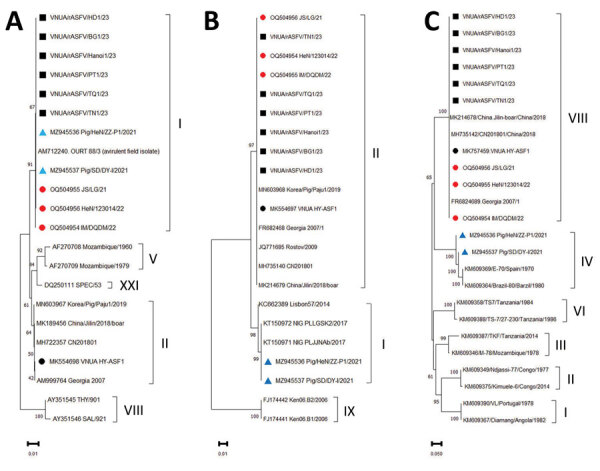
Phylogenetic trees of ASFV, based on the sequences found in the p72 (A), p54 (B), and CD2v (C) regions, Vietnam, 2023. Black squares indicate rASFV I/II strain from Vietnam, red circles indicate rASFV I/II strain from China, black circles indicate first reported ASFV p72 genotype II strain from Vietnam, and blue triangles indicate ASFV p72 genotype I strain from China. Scale bars indicate phylogenetic distance (nucleotide substitutions per site). ASFV, African swine fever virus; rASFV, recombinant ASFV.

Our phylogenetic analysis using MEGAX (https://www.megasoftware.net) revealed that 6 of the 26 samples contained p72 sequences of genotype I, but the p54 sequence belonged to genotype II, and the CD2v sequence belonged to serotype VIII (Table). This finding was consistent with recent pandemic ASFV genotype II viruses (Figure 1), suggesting that those 6 whole blood samples possibly contained recombinant ASFV p72 viruses of genotypes I and II (rASFV I/II). The p72, p54, and CD2v sequences of the remaining viruses were like the viruses from the ASFV genotype II viruses reported in Vietnam (*8*). We inoculated the 6 whole blood samples onto primary porcine alveolar macrophages obtained from the lungs of 8–10-week-old healthy pigs, and we monitored the cultures daily for 5–7 days for hemadsorption. We obtained ASFV isolates positive for hemadsorption from all 6 samples (Appendix Figure 2).

**Table:** Characteristics of 6 rASFV I/II isolates obtained from infected swine on family farms that contained p72 sequences of ASFV genotype I, p54 sequences belonging to genotype II, and the CD2v sequence belonging to serotype VIII, according to phylogenetic analysis, northern Vietnam, 2023*

No.	Isolate name	Collection date	Province	Sample type	Cycle threshold
1	VNUA/rASFV/HD1/23	2023 Sep 23	Hai Duong	Whole blood	20.31
2	VNUA/rASFV/BG1/23	2023 Oct 6	Bac Giang	Whole blood	17.25
3	VNUA/rASFV/Hanoi1/23	2023 Oct 11	Hanoi	Whole blood	17.49
4	VNUA/rASFV/PT1/23	2023 Oct 12	Phu Tho	Whole blood	18.41
5	VNUA/rASFV/TQ1/23	2023 Oct 12	Tuyen Quang	Whole blood	20.41
6	VNUA/rASFV/TN1/23	2023 Oct 13	Thai Nguyen	Whole blood	20.42

*ASFV, African swine fever virus; rASFV, recombinant ASFV.

**Figure 2:**
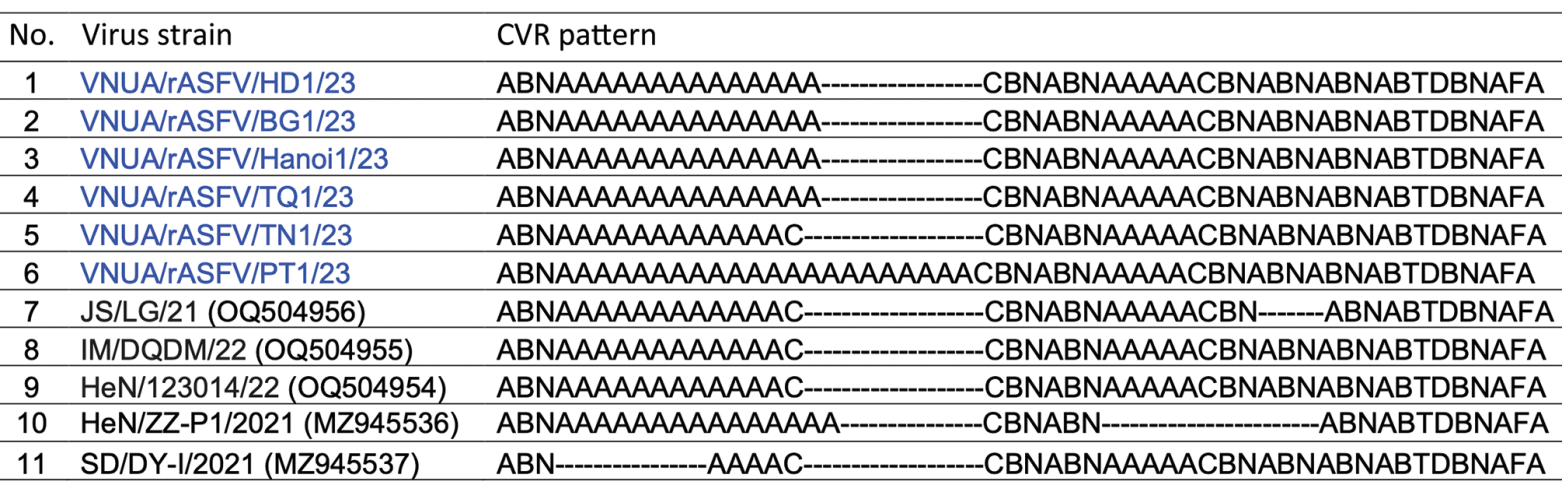
Alignment of ASFV CVR signatures between rASFV genotype I/II strains from Vietnam and China, and genotype I strains from China, recovered from domestic swine in Vietnam and China, 2023. Numbers in parentheses are GenBank accession numbers. ASFV, African swine fever virus; rASFV, recombinant ASFV; CVR, central variable region.

To gain further insight into the genomes of the 6 rASFV I/II isolates, we amplified 10 additional genes (MGF-505–1R, B119L [9GL], I177L, DP96R [UK], A238L, A137R, MGF 360–12L, I226R, B602L, and IGR [between I73R and I329L]) from all 6 isolates by using previously reported and newly designed primers, and we sequenced by using Sanger sequencing (Appendix Table 1) (*12*–*14*). All of the gene sequences we obtained were deposited into GenBank (accession nos.: p72, OR999183–88; p54, OR999177–82; CD2v, OR999147–52; B119L, OR999135–40; DP96R, OR999153–58; B602L, OR999141–46; I177L, OR999159–64; MGF 505–1R, OR999171–76; A238L, PP464965–70; A137R, PP464971–76; MGF 360–12L, PP464977–82; I226R, PP464983–88; and IGR, OR999165–70). Our genetic analysis showed that all target gene sequences of the 6 Vietnamese rASFV I/II isolates matched at the nucleotide level with the corresponding gene sequences of the 3 rASFV I/II strains (JS/LG/21, HeN/123014/22, and IM/DQDM/22) previously reported in China, with the exception of the central variable region (CVR) (Appendix Table 2). The nucleotide sequences of the CVR region of all 3 rASFV I/II strains from China showed a 96-nucleotide insertion compared with the genotype I strain pig/SD/DY-I/2021 isolated from China. The isolate VNUA/rASFV/TN1/23 from Thai Nguyen Province showed the same 96-nucleotide insertion in the CVR region (Appendix Figure 3, panel A). However, the unique 36-nucleotide deletion observed in the CVR region of the JS/LG/21 strain from China was not detected in any of the rASFV I/II strains from Vietnam (Appendix Figure 3, panel B). Four rASFV I/II isolates from Vietnam (VNUA/rASFV/HD1/23, VNUA/rASFV/BG1/23, VNUA/rASFV/Hanoi1/23, and VNUA/rASFV/TQ1/23) had an additional 12-nucleotide insertion (108 nucleotides in total) in the CVR region. The Vietnam isolate VNUA/rASFV/PT1/23 from Phu Tho Province had an additional 108-nucleotide insertion (204 nucleotides in total) in the CVR region (Appendix Figure 3, panel A). Those differences were reflected in the CVR profile of the viruses (Figure 2).

## Conclusions

We report the discovery of 6 rASFV I/II strains from northern Vietnam. To characterize the virus strains, while attempting to sequence the whole genome of the 6 isolates, we rapidly sequenced 13 target genes of these 6 isolates and compared their nucleotide sequences with the corresponding sequences of rASFV I/II strains from China. All sequences, except for the CVR region, matched with the corresponding sequences of rASFV I/II strains from China. The observation of 3 different CVR variants in Vietnam indicates 3 possible independent introductions of rASFV I/II strains into Vietnam. Because ASFV p72 genotype I strains have not been reported in Vietnam, the rASFV I/II strains detected in Vietnam may have originated from China. 

Two live-attenuated ASFV vaccines are now licensed in Vietnam. Both vaccines have been proven to protect young pigs against highly pathogenic ASFV p72 genotype II strains circulating in Vietnam. However, the rASFV I/II strains detected in China appear to be resistant to the live-attenuated ASFV p72 genotype II vaccine candidates (*7*). Therefore, the newly discovered rASFV I/II strains in Vietnam are likely resistant to the immunity induced by the current live ASFV vaccines and could replace the ASFV p72 genotype II strains circulating in the region. This possibility poses a major challenge for disease control and vaccine development and emphasizes the need for increased vigilance in the global control of ASFV.

Appendix Additional information on detection of recombinant African swine fever virus strains of p72 genotypes I and II in domestic pigs, Vietnam, 2023.
