# Supplementary material for: Detection of Recombinant African Swine Fever Virus Strains of p72 Genotypes I and II in Domestic Pigs, Vietnam, 2023
Source: Emerg Infect Dis. 2024 May;30(5):991–4. doi: 10.3201/eid3005.231775 (PMC11060461; doi:10.3201/eid3005.231775)
Supplement: Appendix — Additional information on detection of recombinant African swine fever virus strains of p72 genotypes I and II in domestic pigs, Vietnam, 2023. [file 23-1775-Techapp-s1.pdf]

Article DOI: https://doi.org/10.3201/eid3005.231775

EID cannot ensure accessibility for supplementary materials supplied by authors. Readers who have difficulty accessing supplementary content should contact the authors for assistance.

Detection of Recombinant African Swine Fever Virus Strains of p72 Genotypes I and II in Domestic Pigs, Vietnam, 2023

Appendix

Appendix Table 1. Information of primers used in this study.

No.	Name	Sequence	Length (bp)	Gene	Reference
1	P72.U	GGCACAAGTTCGGACATGT	478	B646L	(*1*)
	P72.D	GTACTGTAACGCAGCACAG			
2	PPA722	CGAAGTGCATGTAATAAACGTC	676	E183L	(*2*)
	PPA89	TGTAATTTCATTGCGCCACAAC			
3	CD2v-1F	GTCGGGGCTACAATCCTTTTATC	690	EP402R	Designed in this study
	CD2v-1R	AAGTGTTGTTTCATTAGATGTAC			
	CD2v-2F	GCTACTCCCCCAAATATCACAT	847		
	CD2v-2R	GTTCTCGATGATCTGCTACTAG			
4	B602L-87070F	GTGGGGTTTGGGTAATTGCATCAA	803–900	B602L	Designed in this study
	B602L-87872F	GCCTTCCTATTCAAAACCTACCC			
5	9GL/F	TAGAGATGACCAGGCTCCAA	357	B119L	(*3*)
	9GL/R	GTTGCATTGGGGACCTAAATACT			
6	MGF	GAGGATGATTTGCCCTTCACTCA	422	MGF 505–1R	(*4*)
	MGR	CGCCACTAGTAAACATTGTTCTATCT			
7	I177L-F	TAGCTTGCCGGTAATGGCTAT	551	I177L	Designed in this study
	I177L-R	TGCGACTCAAGGCAACAT			
8	UK-F	GTTGTCGTGGATAATGCACC	210	DP96R	(*4*)
	UK-R	GGATGGAGCGCATTAGGGAT			
9	ECO1A	CCATTTATCCCCCGCTTTGG	356	Intergenic region between I73R and I329L genes	(*5*)
	ECO1B	TCGTCATCCTGAGACAGCAG			
10	A238L-F	GCGACAATCTTACGTCATGA	1082	A238L	Designed in this study
	A238L-R	CCAAGAATTACCGCACATATG			
11	A137R-F	CCACGTATAGCAACCTATATG	631	A137R	Designed in this study
	A137R-R	CATGAGTTATTGGATGACCTCG			
12	MGF360–12L-F	GCGGCCGAAACATTATTCTTAC	1238	MGF 360–12L	Designed in this study
	MGF360–12L-R	CATACTTGGCAGAATGCCAGC			
13	I226R-F	GATAATGATACCACATGCAT	798	I226R	Designed in this study
	I226-R	TCGATGAGCCATCCACGATA			

References

1. Bastos ADS, Penrith ML, Crucière C, Edrich JL, Hutchings G, Roger F, et al. Genotyping field strains of African swine fever virus by partial p72 gene characterisation. Arch Virol. 2003;148:693–706. PubMed https://doi.org/10.1007/s00705-002-0946-8

2. Gallardo C, Mwaengo DM, Macharia JM, Arias M, Taracha EA, Soler A, et al. Enhanced discrimination of African swine fever virus isolates through nucleotide sequencing of the p54, p72, and pB602L (CVR) genes. Virus Genes. 2009;38:85–95. PubMed https://doi.org/10.1007/s11262-008-0293-2

3. O'Donnell V, Holinka LG, Krug PW, Gladue DP, Carlson J, Sanford B, et al. African swine fever virus Georgia 2007 with a deletion of virulence-associated gene 9GL (B119L), when administered at low doses, leads to virus attenuation in swine and induces an effective protection against homologous challenge. J Virol. 2015;89:8556–66. PubMed https://doi.org/10.1128/JVI.00969-15

4. O'Donnell V, Risatti GR, Holinka LG, Krug PW, Carlson J, Velazquez-Salinas L, et al. Simultaneous deletion of the 9GL and UK genes from the African swine fever virus Georgia 2007 isolate offers increased safety and protection against homologous challenge. J Virol. 2016;91:e01760–16. PubMed

5. Rodriguez JM, Salas ML, Viñuela E. Genes homologous to ubiquitin-conjugating proteins and eukaryotic transcription factor SII in African swine fever virus. Virology. 1992;186:40–52. PubMed https://doi.org/10.1016/0042-6822(92)90059-X

Appendix Table 2. Genotype (based on the genes from the current pandemic p72 genotype II virus and low-virulent genotype I viruses reported in China) and percentage identity of the amplified genes of the Vietnamese rASFV I/II strains compared to those of the rASFV I/II strain JS/LG/21 from China. rASFV I/II and ASFV genotype II strains from Vietnam are highlighted in gray.

		Genotype/Percentage (%) Identity																									
No.	Virus strain	B646L (P72)		B602L (CVR)		B119L (9GL)		A238L		A137R		E183L (P54)		EP402R (CD2v)		DP96R (UK)		I177L		MGF 505–1R		IGR		MGF 360–12L		I226R	
1	VNUA/rASFV/HD1/23	I	100	I	90.94	I	100	I	100	I	100	II	100	II	100	II	100	II	100	II	100	II	100	II	100	II	100
2	VNUA/rASFV/BG1/23	I	100	I	90.94	I	100	I	100	I	100	II	100	II	100	II	100	II	100	II	100	II	100	II	100	II	100
3	VNUA/rASFV/Hanoi1/23	I	100	I	90.94	I	100	I	100	I	100	II	100	II	100	II	100	II	100	II	100	II	100	II	100	II	100
4	VNUA/rASFV/PT1/23	I	100	I	77.47	I	100	I	100	I	100	II	100	II	100	II	100	II	100	II	100	II	100	II	100	II	100
5	VNUA/rASFV/TQ1/23	I	100	I	90.94	I	100	I	100	I	100	II	100	II	100	II	100	II	100	II	100	II	100	II	100	II	100
6	VNUA/rASFV/TN1/23	I	100	I	93.15	I	100	I	100	I	100	II	100	II	100	II	100	II	100	II	100	II	100	II	100	II	100
7	rASFV HeN/123014/22	I	100	I	93.33	I	100	I	100	I	100	II	100	II	100	II	100	II	100	II	100	II	100	II	100	II	100
8	rASFV IM/DQDM/22	I	100	I	93.33	I	100	I	100	I	100	II	100	II	100	II	100	II	100	II	100	II	100	II	100	II	100
9	HeN/ZZ-P1/2021	I	100	I	73.33	I	100	I	100	I	100	I	96.42	I	78.05	I	99.05	I	96.88	I	0	I	86.5	I	0	I	98.09
10	SD/DY-I/2021	I	100	I	75.56	I	100	I	100	I	100	I	96.42	I	78.05	I	99.05	I	96.88	I	0	I	86.5	I	0	I	98.09
11	VN/HY-ASFV1(2019)	II	99.07	II	42.65	II	99.44	II	98.26	II	99.28	II	100	II	100	II	100	II	100	II	100	II	100	II	100	II	100

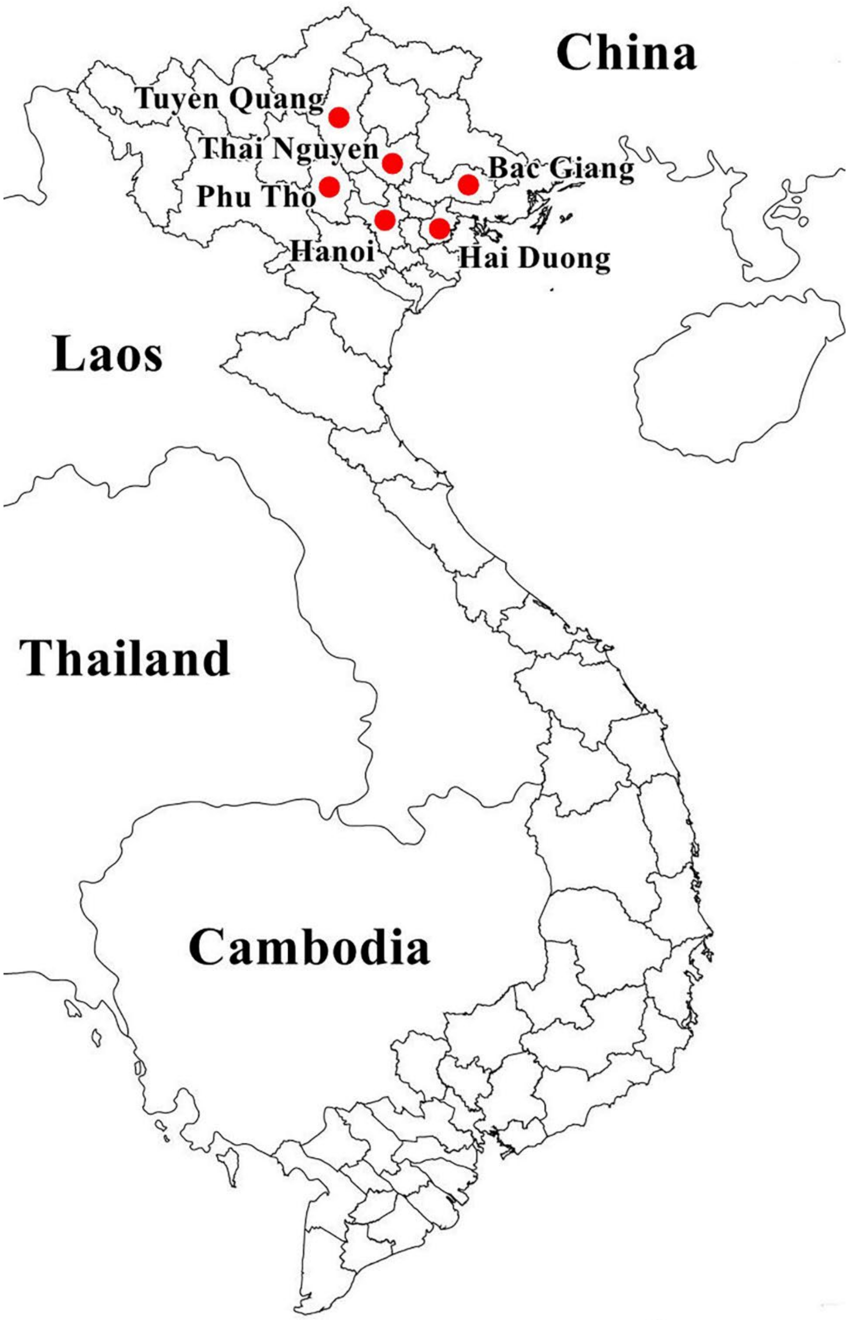

Appendix Figure 1. Map of Vietnam showing the provinces from which rASFV I/II samples were collected.

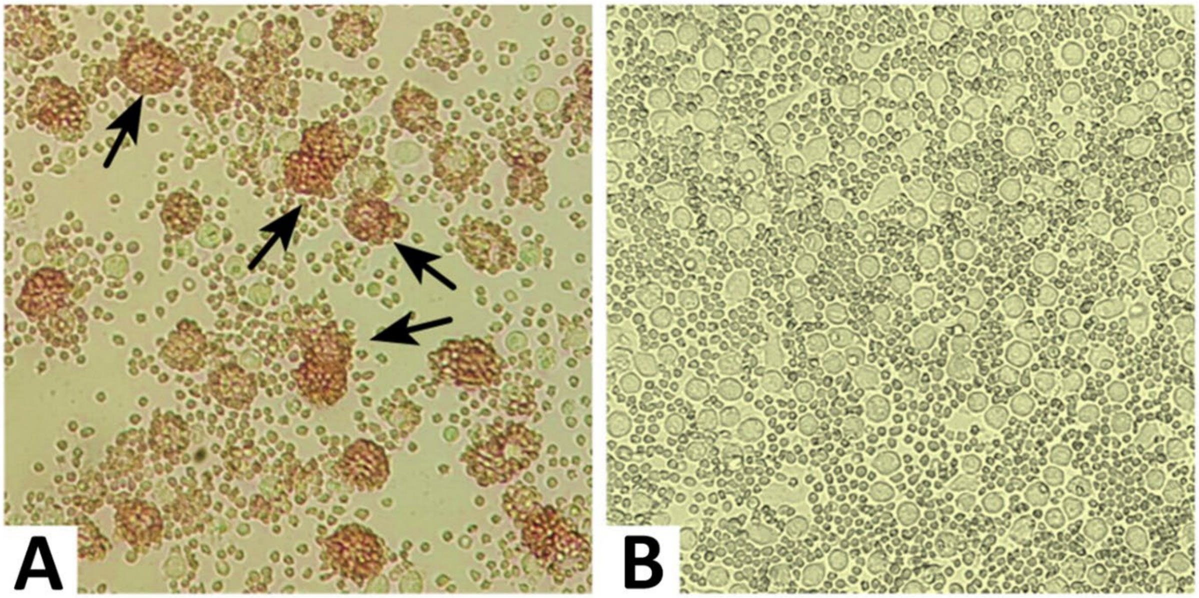

Appendix Figure 2. Isolation of Vietnamese rASFV I/II strains on porcine alveolar macrophage (PAM) cells. Infected (A) and Un-infected (B) PAM cells with Vietnamese rASFV strains. Black arrows indicate hemadsorption (HAD), in which ASFV-infected PAM cells adsorb porcine red blood cells (RBCs).

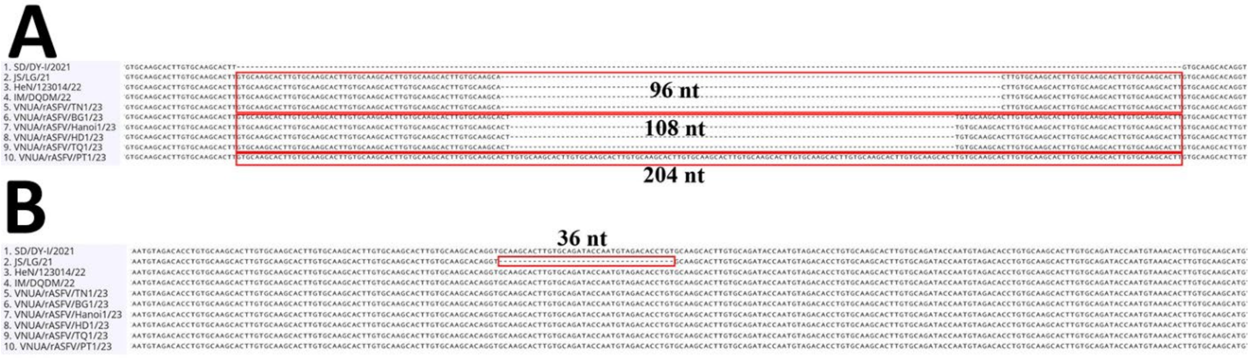

Appendix Figure 3. Comparison of nucleotide (nt) sequences based on the CVR of the B602L gene between Vietnamese rASFV I/II strains and reference strains. A: insertion of 96, 108, and 204 nt; B: deletion of 36 nt.
